# Diagnostic accuracy of standardised qualitative sensory test in the detection of lumbar lateral stenosis involving the L5 nerve root

**DOI:** 10.1038/s41598-017-10641-2

**Published:** 2017-09-06

**Authors:** Jiann-Her Lin, Yi-Chen Hsieh, Yi-Chen Chen, Yun Wang, Chih-Cheng Chen, Yung-Hsiao Chiang

**Affiliations:** 1 PhD Program for Neural Regenerative Medicine, College of Medical Science and Technology, Taipei Medical University and National Health Research Institutes, Taipei, Taiwan; 2 Department of Neurosurgery, Taipei Medical University Hospital, Taipei, Taiwan; 3 Division of Neurosurgery, Department of Surgery, School of Medicine, College of Medicine, Taipei Medical University, Taipei, Taiwan; 4 Institute of Biomedical Sciences, Academia Sinica, Taipei, Taiwan

## Abstract

Misdiagnosis of symptomatic lumbar lateral stenosis (LS) may result in an unfavourable prognosis after surgical treatment. This study investigated the diagnostic accuracy of a standardised qualitative sensory test (SQST) in the detection of symptomatic LS in patients who had degenerative spinal disorders involving the L5 spinal nerve. We prospectively identified 75 patients, of which 60 met the inclusion criteria. Lateral recess stenosis at the L5 level or foraminal stenosis at the L5/S1 level on MRI was identified and graded by a neurosurgeon blinded to any clinical information. The reference criteria for the diagnosis of symptomatic LS were grade III LS on MRI and relevant clinical symptoms. Cutaneous sensory functions of the L5 dermatome on the symptomatic side were evaluated using the SQST. Each item of the SQST showed a satisfactory performance in the diagnosis of LS (sensitivity = 0.455–0.727, specificity = 0.868–1.0). A stepwise selection model identified low-strength von-Frey, high-strength von-Frey, and vibration as the most accurate predictors of symptomatic LS with an area under the receiver operating characteristic curve of 0.9563 (95% confidence interval = 0.9003–1.0). In combination with MRI, the SQST is a promising diagnostic tool for detecting symptomatic LS involving L5 nerve roots.

## Introduction

Degenerative lumbar spinal stenosis (LSS), the most common cause for lumbar spinal surgery in patients older than 65 years^1^, is defined as “buttock or lower extremity pain, which may occur with or without low back pain, associated with a diminished space available for neural and vascular elements in the lumbar spine, secondary to degenerative lumbar spine diseases”^2, 3^. On the basis of anatomy, degenerative LSS can be classified as central stenosis (CS) and lateral stenosis (LS)^4^. Lumbar LS can easily be overlooked, especially when there is coexisting lumbar CS, and misdiagnosis of symptomatic LS may result in an unfavourable prognosis after surgical treatment^5, 6^. Because patients with LS and CS usually have similar clinical presentations^7, 8^, differentiating LS from CS on the basis of only clinical symptoms or signs is difficult. A prospective randomised study conducted in 1995 showed no differences in symptoms, neurological findings, or signs between LS and CS^8^. Since then, few studies have examined differences in clinical presentations between LS and CS. Only in 2014 did a comparative study report that leg pain at rest was more severe in LS than in CS^7^.

In contrast to CS, LS involves the dorsal root ganglia and spinal nerves, which are more vulnerable to compression and produce more severe neuropathic pain^9–13^. Neuropathic pain is redefined as “pain arising as a direct consequence of a lesion or disease affecting the somatosensory system”; this redefinition was suggested by the International Association for the Study of Pain Special Interest Group on Neuropathic Pain in 2008. A substantial proportion of patients with chronic low back pain experienced a neuropathic pain component^14–16^, and the most frequently reported symptoms included hypoesthesia, allodynia, and radiating pain^17^. Standardised qualitative sensory tests (SQSTs) described in the Standardised Evaluation of Pain (StEP)^18^ were validated to detect the neuropathic component of low back pain^19^.

On the basis of the findings of the aforementioned studies, we hypothesised that patients with symptomatic LS have a higher incidence of abnormal SQST results than do those without symptomatic LS, and an SQST combined with magnetic resonance imaging (MRI) can help physicians in the diagnosis of symptomatic LS. This study investigated the diagnostic accuracy of an SQST in the detection of symptomatic LS in patients who had degenerative spinal disorders at the L4/5 or L5/S1 level with disabling back and leg pain.

## Methods

### Design

This was a cross-sectional diagnostic study. All data were prospectively collected at our hospital from April 2016 to February 2017. This study was approved by the Taipei Medical University Joint Institutional Review Board (N201602059). All procedures in this study were performed in accordance with relevant guidelines and regulations, and informed consent was obtained from all participants or their legal guardians.

### Study population

We identified patients older than 18 years who had disabling back pain with or without leg pain and were admitted to our hospital for lumbar spinal surgeries under the care of a single surgeon. Patients who met the following criteria were included: (1) back pain with or without leg pain lasting for more than 3 months after conservative treatment and (2) a corresponding lesion on MRI, such as (a) central spinal stenosis at the L4/5 level due to spondylolisthesis or disc herniation, (b) lateral recess stenosis at the L5 level, (c) foraminal stenosis at the L5/S1 level, or (d) segmental instability requiring surgical treatment at the L4/5 level. We excluded patients who had received a diagnosis of a spinal tumour or infection, presented with cauda equina syndrome, refused to undergo the SQST, had a visual analogue scale (VAS) score of less than 2 for leg pain or soreness, or had simultaneous involvement of L5 nerve roots on both sides. The SQST was designed to examine differences between the symptomatic and contralateral sides; thus, patients with bilateral L5 nerve root involvement may have normal SQST results. To particularly focus on the L5 nerve, we excluded patients who had only L4/5 foraminal stenosis or L5/S1 CS and those with a pathology not involving the L4/5 or L5/S1 level.

### Baseline measures

The SQST was performed according to the standard protocol described in the StEP^18^, except that we used 10 °C for the cold test. In our preliminary study, patients felt a clear cold sensation on their dorsal feet only until the temperature of water was less than 10 °C in a closed room with a temperature of 24–27 °C, and at 10 °C, they could clearly report a difference between the two sides if there was a sensory disturbance. When the temperature of water was 15–20 °C, they felt moderately cold and could hardly identify the difference in cold sensation between the two sides, even when other sensory tests strongly suggested a sensory disturbance. All tests were performed in a quiet room with the temperature maintained at 24–27 °C, and the patients were asked to sit on a chair with their naked feet comfortably situated on a leg rest. The skin of their medial dorsal foot was regarded as the L5 dermatome and the test area. This area was suggested as the L5 dermatome by a study combining clinical symptoms, electromyography, and imaging and surgical findings^20^. A complete SQST comprised eight sensory tests. For each sensory test, an abnormal finding was defined as decreased sensation or hyperalgesia on the symptomatic side compared with the contralateral side, whereas a normal finding was defined as the same sensation between both sides. An investigator (a physician assistant, the third author) interviewed all patients and performed the SQST. Before this study, two investigators (a neurosurgeon, the first author, and the physician assistant, the third author) evaluated the interrater and intrarater agreements of 18 patients. The interrater agreement of each item of the SQST and SLRT was 0.6–1, and the intrarater agreement was 0.7692–1 (Supplemental Table 1).

**Table 1:** Comparison of lateral stenosis(+) and lateral stenosis(−) groups.

Stenosis type	Lateral Stenosis		*p*
	With	Without	
n	22	38	
Age	56.45 ± 15.5	66.26 ± 10.9	0.0920
Gender			
*F*	10	28	0.0500
*M*	12	10	
Diagnosis			
*Disc herniation*	12	10	0.1027
*Spondylolisthesis*	9	25	
*Spondylosis*	2	4	
Back pain	5.32 ± 3.22	5.84 ± 3.41	0.9982
Back soreness	4.29 ± 3.81	4.54 ± 3.45	0.1967
Leg pain	7.27 ± 2.68	5.00 ± 3.65	0.9982
Leg soreness	2.57 ± 3.57	4.00 ± 3.18	0.9314
SF36			
*Physical Functioning*	24.8 ± 16.6	17.39 ± 13.5	0.6381
*Role-Physical*	30.44 ± 7.08	29.42 ± 5.43	0.9982
*Role-Emotional*	36.69 ± 9.66	36.6 ± 9.75	0.9982
*Bodily Pain*	25.79 ± 10.5	26.35 ± 7.89	0.9982
*Vitality*	41.16 ± 10.8	38.88 ± 9.64	0.9965
*Mental Health*	45.5 ± 10.9	41.96 ± 11.6	0.9760
*Social Functioning*	35.7 ± 13.1	33.43 ± 12.4	0.9982
*General Health*	42.37 ± 10.2	40.98 ± 11.4	0.9982
*AGG_Physics*	21.57 ± 11.8	18.95 ± 9.72	0.9950
*AGG_Mental*	47.38 ± 9.91	45.61 ± 10.9	0.9982
ODI	23.43 ± 9.68	21.67 ± 6.46	0.9978
JOA	16.79 ± 4.76	17.53 ± 3.77	0.9982

#### Lumbar spine MRI

All lumbar spine MRI examinations were performed using a 1.5 T MR scanner (Signa, GE Healthcare, Waukesha, WI, USA) with a traditional cervical–thoracic–lumbar spine coil (Signa, GE Healthcare) at the study hospital. The patients were placed in a supine position with a cushion under both knees, and T2-weighted fast spin-echo axial and sagittal images were obtained (repetition time/echo time, 3000.00/110.00 ms and 3050.00/110.00 ms; field of view, 18 cm and 30 cm; matrix, 320°–224° and 384°–214°; echo train length, 20 and 25; excitations, 3–4 and 4 for axial and sagittal scans, respectively; slice thickness, 4 mm; slice gap, 0.4 mm; and flip angle, 90°).

### Reference criteria: Symptomatic LS

**Figure 1:**
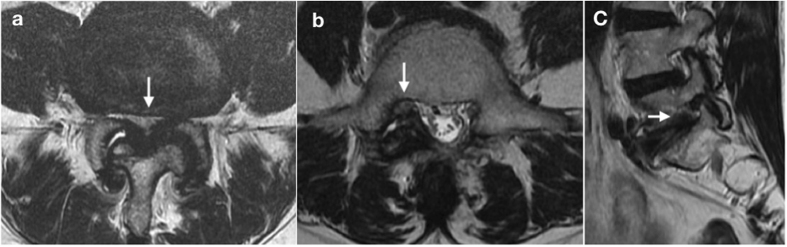
The arrows indicate Schizas’s grade D central stenosis (**a**) and grade III lateral recess stenosis (**b**) on axial T2-weighted magnetic resonance images, and grade III foraminal stenosis (**c**) on a sagittal T2-weighted magnetic resonance image.

The reference criteria for the diagnosis of symptomatic LS were a grade III LS on MRI and clinical symptoms (radiating or shooting pain below the knee or L5 dermatomal pain distribution). A neurosurgeon (the first author) graded the LS and CS of each patient and then classified each patient as having LS or CS or no stenosis based on MRI findings. This observer was unaware of the patient’s medical history or the results of SQSTs. The observer had 8 years of postresidency experience in reading spinal MRIs. CS was defined as central spinal stenosis at the L4/5 level caused by spondylolisthesis or disc herniation and was graded according to Schizas’s and Lee’s classification^21, 22^. CS was regarded to be present if the grade was B, C, or D according to Schizas’s classification (Fig. 1). LS was defined as lateral recess stenosis at the L5 level or foraminal stenosis at the L5/S1 level^23^ (Fig. 1). Lateral recess stenosis was graded according to Bartynski’s classification^24^, and foraminal stenosis was graded following Lee’s classification^25^. LS diagnosis was a radiological diagnosis based only on imaging grading; however, diagnosis of symptomatic LS was a clinical diagnosis based on both imaging grading and clinical symptoms. LS was regarded as present if the grade of lateral recess stenosis or foraminal stenosis was 3. In addition, the diagnosis of symptomatic LS in this study was made by a senior neurosurgeon who had 40 years of experience in the management of degenerative lumbar stenosis based on clinical symptoms (radiating or shooting pain below the knee or L5 dermatomal pain distribution) and corresponding grade III lateral recess stenosis or grade III foramina stenosis on MRI. In this study, the foraminal stenosis or lateral recess stenosis of patients with symptomatic LS was surgically confirmed, and their leg pain improved immediately after the surgery.

### Clinical information collection

We prospectively collected the data of all patients, including age, sex, diagnosis, results of the preoperative Short Form-36 (SF-36; Chinese version)^26^, the Japanese Orthopedic Association (JOA) score for low back pain^27^, the Oswestry disability index (ODI; Chinese version)^28^, and VAS scores for leg pain or soreness, or back pain or soreness (the scale ranged from 0 to 10, with 0 indicating “no pain at all” and 10 indicating “the most imaginable pain”). We regarded soreness as a discomfort in patients with symptomatic LS.

### Statistical analysis

Data are presented as the mean ± standard deviation. Comparisons between continuous variables (age, SF-36, ODI, and JOA) were performed using the Student *t* test, one-way analysis of variance (ANOVA), and the Mann–Whitney U test. Comparisons between categorical variables (sex, level, diagnosis, stenosis grade, and SQST results) were performed using the chi-square test. The Holm–Sidak correction method was used for multiple comparisons. Logistic regression models were used to evaluate the receiver operating characteristic (ROC) curve for various sensory and test assessments to identify LS. The areas under the curves (AUCs) indicated the highest possible sensitivity and specificity. A stepwise model selection method was used to confirm logistic regression as the best method to build a predictive model. Statistical analyses were performed using Prism 7 for Mac (GraphPad Software, La Jolla, CA, USA) and SAS software, version 9.4 (SAS Institute, Cary, NC).

### Availability of materials and data

The datasets generated and/or analysed during the current study are available from the corresponding author on reasonable request.

## Results

**Figure 2:**
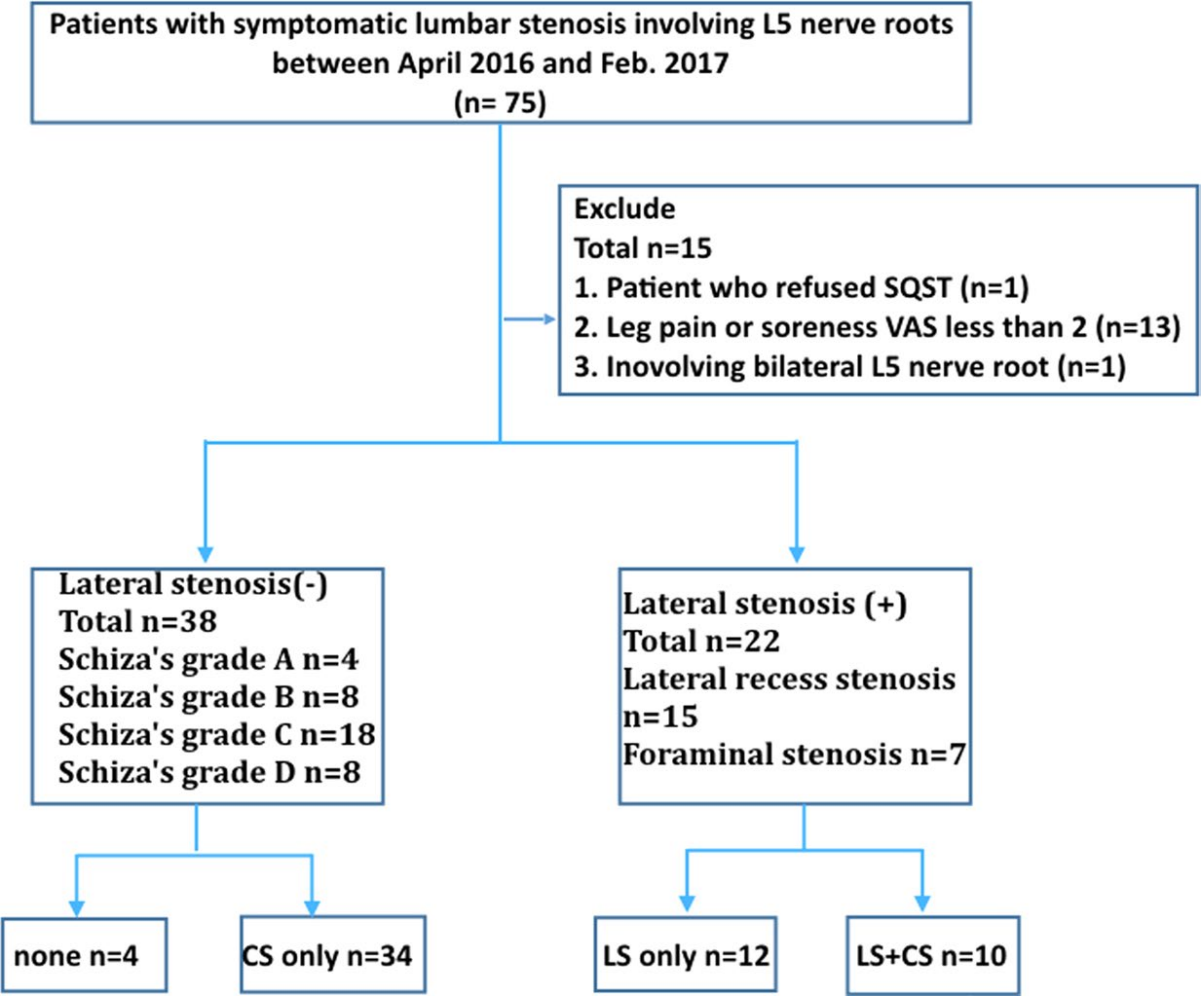
Flowchart of the inclusion of eligible patients. CS, central stenosis; LS, lateral stenosis; SQST, standardised qualitative sensory test.

Between April 2016 and February 2017, 75 patients were assessed for eligibility (Fig. 2). Of these patients, 15 were excluded for the following reasons: 1 patient refused to undergo the SQST, 13 had a VAS score of less than 2 for leg pain or soreness, and 1 had bilateral L5 nerve root involvement. Finally, 60 patients were included. According to the presence of symptomatic LS (grade III LS on MRI and patients’ clinical symptoms), 22 and 38 patients were included into the LS(+) and LS(−) groups, respectively. In the LS(+) group, 12 patients had only LS and 10 had both CS and LS. By contrast, in the LS(−) group, 34 patients had only CS, and 4 had no stenosis. No significant difference was observed between the LS(+) and LS(−) groups in age, diagnosis, back pain, back soreness, leg pain, leg soreness, SF-36 score, ODI, or JOA score, but a difference was observed in sex (Table 1). In the subgroup analysis, no significant difference was observed among the four groups (Supplementary Table S2). In the LS(+) group, 15 patients had lateral recess stenosis (graded as 3) and 7 patients had foraminal stenosis (graded as 3), whereas in the LS(−) group, the stenosis of 4, 8, 18, and 8 patients was graded as A, B, C, and D, respectively, according to Schizas’s classification (Table 2). The stenosis type significantly differed between the LS(+) and LS(−) groups (Table 2). Kappa values for the intrarater and interrater agreements of the SQST are listed in Supplementary Table S1, and the agreement of all items was good to excellent. ncidence of sensory disturbance in each item of the SQST in patients with LS than in patients without LS (73% vs. 8% for low-strength von-Frey, *p* < 0.0001; 73% vs. 3% for high-strength von-Frey, *p* < 0.0001; 55% vs. 0% for pinprick, *p* < 0.0001; 45% vs. 0% for brush, *p* < 0.0001; 59% vs. 3% for blunt, *p* < 0.0001; 59% vs. 8% for vibration, *p* < 0.0001; 68% vs. 3% for warmth, *p* < 0.0001; and 73% vs. 16% for cold, *p* < 0.0001; Supplement Table 3).

**Table 2:** Grading of central stenosis and lateral stenosis in lateral stenosis(+) and lateral stenosis(−) groups.

		Lateral stenosis		*p*
		Yes	No	
Central stenosis (L4/5 spinal canal)		22	38	
*Schiza*				0.0012
A		12	4	
B		5	8	
C		3	18	
D		2	8	
*Lee*				0.0045
0		9	4	
1		5	4	
2		3	4	
3		5	26	
Lateral stenosis				
*L5 lateral recess*				<0.0001
0		3	14	
1		1	12	
2		3	12	
3		15	0	
*L5/S1 foraminal stenosis*				0.0027
0		11	25	
1		1	5	
2		3	8	
3		7	0

**Table 3:** AUC, sensitivity, specificity, PPV, and NPV of the SQST in the detection of lateral stenosis.

Variables	AUC	Sensitivity	Specificity	PPV	NPV
Low Strength von frey	0.82 (0.72–0.93)	0.82 (0.72–3.93)	0.92 (0.84–1.01)	0.84 (0.68–1.01)	0.85(0.75–0.96)
High Strength von frey	0.85 (0.75–0.95)	0.73 (0.54–0.91)	0.97 (0.92–1.03)	0.94 (0.83–1.05)	0.86 (0.76–0.96)
Pinprick	0.77 (0.67–0.88)	0.55 (0.34–0.75)	1.00 (1.00–1.00)	1.00 (1.00–1.00)	0.79 (0.68–0.91)
Brush	0.73 (0.62–0.83)	0.46 (0.25–0.66)	1.00 ((1.00–1.00)	1.00 (1.00–1.00)	0.76 (0.76–0.88)
Blunt	0.78 (0.67–0.89)	0.59 (0.39–0.80)	0.97 (0.92–1.03)	0.93 (0.79–1.06)	0.80 (0.69–0.99)
Vibration	0.76 (0.64–0.87)	0.59 (0.39–0.80)	0.92 (0.94–1.01)	0.81 (0.62–1.00)	0.80 (0.68–0.92)
Warm	0.83 (0.73–0.93)	0.68 (0.49–0.88)	0.97 (0.92–1.03)	0.94 (0.82–1.06)	0.84 (0.73–0.95)
Cold	0.73 (0.61–0.85)	0.59 (0.39–0.80)	0.87 (0.76–0.98)	0.72 (0.52–0.93)	0.79 (0.66–0.91)

AUC, area under the curve; PPV, positive predictive value; NPV, negative predictive value; SQST, standardised qualitative sensory test; SLRT, straight leg rising test. Corresponding 95% confidence intervals are in parentheses.

**Figure 3:**
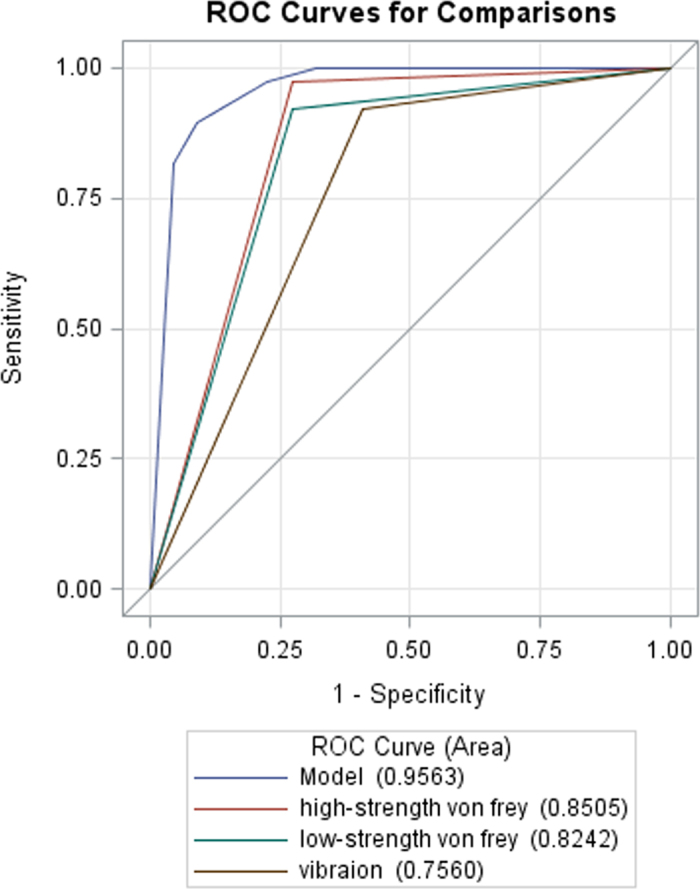
Low-strength von-Frey, high-strength von-Frey, and vibration were identified to be the most accurate predictors of lateral stenosis through a stepwise selection procedure with an area under the receiver operating characteristic curve of 0.9563 (95% confidence interval = 0.9003–1.0).

Table 3 lists the AUC, sensitivity, specificity, positive predictive values, and negative predictive values of the SQST for symptomatic LS. The sensitivity and specificity of the diagnosis of LS on MRI were 72.7% and 92.1% for low-strength von-Frey, 72.7% and 97.4% for high-strength von-Frey, 54.5% and 100% for pinprick, 45.5% and 100% for brush, 59.1% and 97.4% for blunt, 59.1% and 92.1% for vibration, 68.2% and 97.4% for warmth, and 59.1% and 86.8% for cold, respectively. All eight items of the SQST were then chosen in the stepwise selection procedure. The final model identified low-strength von-Frey, high-strength von-Frey, and vibration as the most accurate predictors of LS with an area under the ROC curve of 0.9563 (95% confidence interval = 0.9003–1.0; Fig. 3).

## Discussion

The results of this study demonstrated that among patients with a pathology at the L4/5 or L5/S1 level involving the L5 spinal nerve root, those with symptomatic LS had a higher incidence of abnormal SQST results, and low-strength von-Frey, high-strength von-Frey, and vibration were identified to be the most accurate predictors of symptomatic LS.

The current study is the first to show that the SQST is a useful tool for differentiating patients with symptomatic LS from those with CS. Typically, patients with CS have more pain in the back than in the legs, whereas those with LS have more pain in the legs than in the back. However, the identification of symptomatic LS is difficult when it coexists with CS and when patients have pain in both the back and legs. Therefore, we included only patients who had a VAS of more than 2 for leg pain or soreness in this study. A few studies have compared clinical presentations between LS and CS. Leg pain at rest was reported to be more severe in patients with LS compared with those with CS^7^. In agreement with this finding, we found a trend of leg pain being more severe in patients with LS, but the difference was not statistically significant. However, a prospective randomised trial showed that sensory disturbances did not differ between patients with LS and those with CS^8^. In contrast to this finding, our study results revealed that patients with symptomatic LS had a significantly higher incidence of abnormal SQST results. We believe that the standardised protocol of the SQST in the StEP improved the sensitivity and specificity of the diagnosis of symptomatic LS in our study. Furthermore, symptomatic LS is easily overlooked, particularly when there is coexisting CS. In our study, 10 patients had both CS and LS, and all of them exhibited sensory disturbances in at least one item of the SQST in their affected limb. Patients with both CS and LS had more LS-like clinical presentations. Compared with patients with only CS, more patients with both CS and LS had abnormal findings in SQSTs (Supplementary Table S3), and the SQST is a promising tool in the diagnosis of LS, even when there is coexisting CS.

In our study, patients with symptomatic LS had a higher incidence of abnormal findings in the SQST, which are indicators of neuropathic pain. Neuropathic pain refers to pain arising as a direct consequence of a lesion or disease affecting the somatosensory system^29^. Low back pain may comprise both nociceptive axial and neuropathic radicular pain. In patients with such pain, differentiating between nociceptive and neuropathic pain is clinically important, because these components require different pain management strategies. This definition has been useful to distinguish between neuropathic and other types of pain; however, it lacks both diagnostic specificity and anatomic precision^30–35^. Consequently, a grading system was proposed to provide a more precise definition useful for clinical and research purposes^29, 36^. The proposed grading system is based on the certainty of neuropathic pain and has four criteria:

1. Pain with a distinct neuroanatomically plausible distribution.
2. A history suggestive of a relevant lesion or disease affecting the peripheral or central somatosensory system.
3. The presence of negative or positive neurological signs concordant with the distribution of pain in neurological examination or in the more objective confirmatory tests (quantitative sensory testing or laboratory tests).
4. Demonstration of the relevant lesion or disease by at least one confirmatory test.

The grading of certainty for the presence of neuropathic pain is considered “defined” if all criteria are met, “probable” if both criteria 1 and 2 plus either 3 or 4 are met, and “possible” if only 1 and 2 are met without confirmatory evidence from 3 or 4.

In this study, on the basis of the grading system of neuropathic pain, we modified these four criteria for grading neuropathic pain in lumbar radiculopathy specifically involving the L5 spinal nerve:

1. Leg pain below the knee along or not along the L5 dermatome.
2. A history of low back pain or leg pain.
3. Abnormal findings in the SQST.
4. Stenosis showing the involvement of the L5 dorsal nerve root or dorsal root ganglion on MRI.

Leg pain in patients with stenosis, particularly LS, and abnormal findings in the SQST were classified as definied neuropathic pain (all criteria were satisfied). Leg pain in those with stenosis but with normal findings in the SQST was defined as probable neuropathic pain. Leg pain in those without either stenosis or abnormal findings in the SQST was defined as possible neuropathic pain. Accordingly, leg pain in 100% of patients with symptomatic LS and 18% of patients with CS was definite neuropathic pain. Leg pain in 82% of patients with CS was probable neuropathic pain and that in four patients without LS or CS was possible neuropathic pain. The risk of nerve root injury is higher in LS than in CS because of the anatomic structures. In contrast to the central canal, the lateral canal has a much narrower space; thus, nerve root injury can easily occur in LS. In addition, nerve structures involved in LS and CS are different. In LS, the dorsal root ganglia and their spinal nerves are involved, and injuring these structures caused much severer pain responses in an animal model compared with injury of dorsal roots in the central canal^9–13^. In summary, the relatively narrow anatomical structures and the nerve structures comprising the lumbar lateral canal may underpin the mechanism that explains why patients with symptomatic LS have more abnormal sensory test results and neuropathic pain.

We defined grade 3 as the presence of LS in both the lateral recess and foramen. The grade at which LS causes symptoms remains controversial. Grades II and III were defined as the presence of stenosis in a previous study^24^. In our study, 25% (4/16) of patients with grade II LS and 95.5% (21/22) of patients with grade III LS exhibited abnormal SQST results. Although four patients with grade II LS had abnormal SQST results, their leg pain was not more severe than their back pain, was not in a radiating or shooting manner, and did not extend below the knees. On the basis of their clinical presentations, a diagnosis of symptomatic LS was not established. Accordingly, grade III was defined as the presence of LS, not including grade II. The VAS scores for leg pain of patients with grade 0–1 LS, grade 2 LS, and grade 3 LS were 4.86 ± 3.73, 5.19 ± 3.66, and 7.23 ± 2.68, respectively (one-way ANOVA, p = 0.047). The VAS score for leg pain increased with the grade of LS. Accordingly, the results of the current study showed that grade 3 LS in either the lateral recess or foramen is more likely to result in abnormal sensory test results or leg symptoms.

LS consisted of lateral recess stenosis and foraminal stenosis. A study reported 72% sensitivity of MRI in the detection of Grade II–III lateral recess stenosis related to the degenerative bony changes of the surrounding structure, but it excluded stenosis related to disc protrusion^24^. In other words, the sensitivity of MRI in the detection of lateral recess stenosis related to disc protrusion is not known. Another study reported that the sensitivity, specificity, and accuracy of MRI in the detection of a lumbar disc protrusion is 92%, 91%, and 92%, respectively, which was confirmed by surgical findings^37^. Thus, we believe that MRI can more accurately detect lateral recess stenosis related to disc protrusion compared with that related to degenerative bony changes. In this study, 67% of grade III lateral recess stenosis was related to disc protrusion, and 33% was related to degenerative facet joint hypertrophy or pars interarticularis hypertrophy. Grade III lateral recess stenosis shown on preoperative MRI of all patients was surgically confirmed in our study. In patients with grade 0–II lateral recess stenosis, a disc protrusion or very severe stenosis related to the bony structure was not found at the lateral recess during the operation, but it was very difficult to grade lateral recess stenosis intraoperatively. Thereafter, a bias may have occurred due to our underestimation of the incidence of grade II or III LS related to degenerative bony changes in patients in the LS(−) group. However, for lateral recess stenosis related to disc protrusion, the bias was very limited.

This study has some limitations. Although the number of participants was small, and confidence intervals around the estimates of sensitivity, specificity, and positive and negative predictive values were therefore slightly wide, the power was still satisfactory (87.29%) when we considered the cold sensory test with the smallest ROC curve (0.73). This study included only patients who experienced disabling pain for more than 3 months; thus, the results of the SQST in patients with acute pain would not necessarily be the same. The focus of this study was to identify symptomatic LS. For asymptomatic LS, the SQST was not validated in this study. All patients were recruited from a single hospital, and local factors influencing the incidence of lumbar LS cannot be excluded. Therefore, the generalisability of our results may be limited. The SQST is used to detect differences in sensation between two sides. Thus, it may be not able to detect differences in a patient who has sensory disturbance on both sides. The SQST is a qualitative test, and it cannot reflect how different patients feel between sides. The accuracy of the SQST is based on patients’ responses, and it heavily depends on patients’ understanding and cooperation. This study excluded patients who could not respond correctly to the SQST. In addition, the L5 dermatome may vary among patients. The skin site tested in this study was the medial dorsal foot, which was regarded as the L5 dermatome in a comprehensive study combining clinical symptoms, electromyography, and imaging and surgical findings^20^. However, the medial dorsal foot may not be the L5 dermatome in a few patients. The results of this study should be interpreted with caution. Finally, because MRI has only 72% sensitivity in the detection of grade II–III lateral recess stenosis related to the degenerative bony structure, a bias may have occurred in this study due to the underestimation of the incidence of such stenosis. However, for lateral recess stenosis related to disc protrusion and foraminal stenosis, underestimation was limited.

## Conclusions

The incidence of abnormal sensory test results was higher in patients with symptomatic LS than in those with CS. The results of this study indicated that in combination with MRI, the SQST can serve as a diagnostic tool in the identification of symptomatic LS in patients with symptomatic lumbar stenosis involving the L5 spinal nerve.

## Electronic supplementary material

supplementary tables
