# Supplementary material for: Diagnostic accuracy of standardised qualitative sensory test in the detection of lumbar lateral stenosis involving the L5 nerve root
Source: Sci Rep. 2017 Sep 6;7:10598. doi: 10.1038/s41598-017-10641-2 (PMC5587645; doi:10.1038/s41598-017-10641-2)
Supplement: Supplementary file 1 — supplementary tables [file 41598_2017_10641_MOESM1_ESM.pdf]

Diagnostic accuracy of standardised qualitative sensory test in the detection of lumbar lateral stenosis involving the L5 nerve root

Jiann-Her Lin[1,2,3,4], Yi-Chen Hsieh[1], Yi-Chen Chen[2], Yun Wang[1], Chih-Cheng Chen[4], *Yung-Hsiao Chiang[1,2,3]

1. PhD Program for Neural Regenerative Medicine, College of Medical Science and Technology, Taipei Medical University and National Health Research Institutes, Taipei, Taiwan

2. Department of Neurosurgery, Taipei Medical University Hospital, Taipei, Taiwan

3. Division of Neurosurgery, Department of Surgery, School of Medicine, College of Medicine, Taipei Medical University, Taipei, Taiwan

4. Institute of Biomedical Sciences, Academia Sinica, Taipei, Taiwan

Supplement table 1 intra- and inter-rater agreement of SQST

	Agreement	
	intra-rater	inter-rater
Low-strength von-frey	0.7692	0.7692
High-strength von-frey	0.6400	0.7692
Pinprick	0.7692	0.7692
Brush	0.7692	1.0000
Blunt	0.6842	0.8525
Vibration	1.0000	1.0000
Warm	0.6000	0.7692
Cold	0.6000	0.8235

Supplement table 2 The demographics and subgroup analysis

Stenosis type	Both	Lateral	Central	None	*p*
n	10	12	34	4	
Age	62.1 ± 15.3	51.75 ± 14.7	66.29 ± 10.4	66.00 ± 16.4	0.2813
Gender					
F	6	4	25	3	0.0914
M	4	8	9	1	
Diagnosis					
Disc herniation	5	7	8	2	0.4231
Spondylolisthesis	4	5	23	2	
Spondylosis	1	1	4	0	
Back pain	5.7 ± 3.09	5 ± 3.41	5.971 ± 3.38	4.75 ± 4.03	0.7926
Back soreness	4.429 ± 3.55	4.143 ± 4.34	4.727 ± 3.46	3 ± 3.37	0.8253
Leg pain	7.5 ± 1.9	7.083 ± 3.26	5.088 ± 3.63	4.25 ± 4.35	0.0985
Leg soreness	4.143 ± 4.26	1 ± 1.92	4.125 ± 3.3	3 ± 2.00	0.1422
SF36					
Physical Functioning	21.81 ± 20.5	27.3 ± 13.1	16.39 ± 12.5	25.88 ± 20.9	0.1348
Role-Physical	31.51 ± 7.9	29.55 ± 6.53	28.62 ± 3.96	36.29 ± 11	0.0774
Role-Emotional	39.65 ± 11.7	34.21 ± 7.23	36.61 ± 9.99	36.48 ± 8.68	0.639
Bodily Pain	22.92 ± 10.8	28.19 ± 10.1	25.43 ± 7.07	34.18 ± 11.3	0.1374
Vitality	41.6 ± 10.8	40.8 ± 11.3	37.9 ± 8.64	47.24 ± 14.8	0.2829
Mental Health	46.9 ± 14.7	44.33 ± 6.73	41.45 ± 11.5	46.26 ± 13.7	0.5301
Social Functioning	30.41 ± 13.6	40.1 ± 11.5	33.09 ± 11.7	36.37 ± 18.8	0.2734
General Health	40.86 ± 13.3	43.63 ± 7.17	39.91 ± 10.9	50.12 ± 13.3	0.3019
AGG_Physics	18.92 ± 13.9	23.78 ± 9.83	17.98 ± 9.03	27.26 ± 12.9	0.1862
AGG_Mental	48 ± 13.1	46.87 ± 6.84	45.13 ± 10.9	49.68 ± 12.3	0.779
ODI	28.57 ± 9.02	18.29 ± 7.74	22.03 ± 6.52	18.75 ± 5.85	0.0415
JOA	14.71 ± 4.46	18.86 ± 4.38	17.16 ± 3.79	20.5 ± 2.08	0.0856

Supplement table 3 The results of standardized qualitative sensory tests

Sensory disturbance (%)	LS(+)			LS (-)			*p*
	Total	Both	LS	Total	CS	None	
Low-strength von-frey	73%	60%	83%	8%	9%	0%	< 0.0001
High-strength von-frey	73%	50%	92%	3%	3%	0%	< 0.0001
Pinprick	55%	40%	67%	0%	0%	0%	< 0.0001
Brush	45%	30%	58%	0%	0%	0%	< 0.0001
Blunt	59%	40%	75%	3%	3%	0%	< 0.0001
Vibration	59%	70%	50%	8%	9%	0%	< 0.0001
Warm	68%	50%	83%	3%	3%	0%	<0.0001
Cold	73%	60%	83%	16%	18%	0%	<0.0001

LS, lateral stenosis; CS, central stenosis

p value indicated the comparison between LS (+) and LS(-).
